# Charged particle guiding and beam splitting with auto-ponderomotive potentials on a chip

**DOI:** 10.1038/s41467-020-20592-4

**Published:** 2021-01-15

**Authors:** Robert Zimmermann, Michael Seidling, Peter Hommelhoff

**Affiliations:** Department Physik, Friedrich-Alexander-Universität Erlangen-Nürnberg (FAU), Staudtstraße 1, 91058 Erlangen, Germany

**Keywords:** Atom optics, Matter waves and particle beams, Quantum mechanics, Mass spectrometry

## Abstract

Electron and ion beams are indispensable tools in numerous fields of science and technology, ranging from radiation therapy to microscopy and lithography. Advanced beam control facilitates new functionalities. Here, we report the guiding and splitting of charged particle beams using ponderomotive forces created by the motion of charged particles through electrostatic optics printed on planar substrates. Shape and strength of the potential can be locally tailored by the lithographically produced electrodes’ layout and the applied voltages, enabling the control of charged particle beams within precisely engineered effective potentials. We demonstrate guiding of electrons and ions for a large range of energies (from 20 to 5000 eV) and masses (from 5 · 10^−4^ to 131 atomic mass units) as well as electron beam splitting for energies up to the keV regime as a proof-of-concept for more complex beam manipulation.

## Abstract (web summary)

There is interest in controlling particle beams using electric fields and using them in compact devices. Here the authors demonstrate guiding and splitting of charged particle (electron and ion) beams on a chip designed with special structures.

## Introduction

Since the postulation of the wave-like nature of matter^1^ and the formulation of the theory of quantum mechanics in subsequent years, experiments with free electrons showed fundamental concepts, such as like matter-wave (electron) interference^2^, the Aharanov–Bohm effect^3,4^, and antibunching through Hanbury Brown–Twiss experiment^5^. Furthermore, the invention of ion traps^6^, which allowed a previously unseen control over the external degrees of freedom of electrons, enabled high-precision measurements of the electrons mass^7^ and the magnetic moment^8,9^. For quantum information processing, planar chip-based ion traps have become a promising technology, as state-of-the art manufacturing techniques used in semiconductor and printed circuit board technology can be used to create miniaturized traps with many micro-structured electrodes^10–15^. Similarly, electromagnetic potentials customized in this way have been used in quantum manipulation experiments with neutral atoms in magnetic chip traps^15^. Even though guiding and splitting of an electron beam by the effective time-independent (ponderomotive) force created by the microwave field have been demonstrated to work for very low electron energies (<5 eV)^16–18^, quantum mechanical experiments like the coupling of a free electron wave packet into the ground state of the transverse quantum state of the ponderomotive potential or the coherence of the microwave-based beam splitting have yet to be shown. One reason is that, in order for the energy separation of the quantum states of the transverse potential to be large enough to ensure adiabaticity, the driving microwave signal needs to have a frequency in the tens of gigahertz and a power far beyond 100 W to get a trap frequency high enough. This is difficult to realize as heating, propagation of different modes, and impedance matching must be considered^16,17^.

In the following, we describe the electrostatic counterparts of planar electrodynamic microwave traps and beam splitters that dramatically expand the range of trapping parameters, while maintaining the same physical (but not technical) operation principle. These devices can simultaneously confine charged particles with vastly different masses in highly customizable potential landscapes. Further miniaturization can lead to the realization of an electron interferometer based on the ponderomotive guiding and splitting potential. Importantly, the applicable electron energies for these structures are high enough that they can be used in combination with a standard electron microscope, as demonstrated in the electron beam splitting experiment below.

**Fig. 1:**
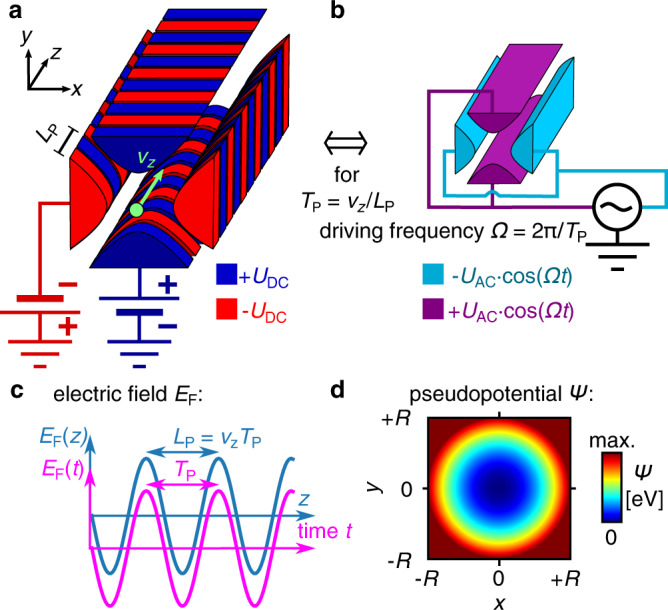
Principle of auto-ponderomotive guiding. **a** When a beam of charged particles with velocity *v*_*z*_ (green arrow) is injected into a structure consisting of electrostatic electrodes with spatially periodic voltages, the charged particle beam will be subjected to the equivalent transverse restoring force as charged particles in a linear electrodynamic trap with the same geometry, but with unsegmented electrodes as shown in **b**. This is because the spatially periodic electrostatic quadrupole field with period length *L*_P_ leads to an alternating field with the periodicity *T*_P_ = *L*_P_/*v*_*z*_ in the rest frame of the moving particles (**c**). Like in a linear Paul trap, the charged particles experience a time-averaged harmonic pseudopotential, the ponderomotive potential, resulting in a restoring force towards the centerline. **d** The pseudopotential *Ψ* of both linear trap realizations shown in **a** and **b** can be made identical. *R* represents the electrodes’ minimal distance from the guide’s center.

Like in undulators in particle accelerators^19^ and similarly in charged particle optics^20–22^, a charged particle beam with well-defined forward velocity is created and injected into a structure consisting of segmented electrodes with spatially alternating direct current (DC) voltages, as illustrated in Fig. 1. The electrostatic potential is transformed into an alternating potential in the rest frame of the moving particles and, thus, the particles are subjected to the same restoring transverse force as they are in a conventional linear Paul trap with alternating current (AC) voltages on non-segmented electrodes. Since the driving frequency generating this ponderomotive force originates from the particles’ forward velocity, we call these devices here “auto-ponderomotive” to distinguish them from their electrodynamic counterparts. Like in a linear Paul trap^6^, the stability of the trajectories of the charged particles is described by the two well-known dimensionless stability parameters *α* and *q*. For nonrelativistic particles, these parameters depend only on the amplitude of the applied voltages, the particle acceleration voltage *U*_A_, and the guide’s geometry. Yet, in stark contrast to electrodynamic traps, the stability parameters are independent of the charge-to-mass ratio (see Supplementary Note 4). In the following, we present two auto-ponderomotive structures, one to show guiding over a curved path and another one for beam splitting, and discuss their potential applications in future quantum mechanical experiments based on their superior performance compared to the above mentioned planar microwave structures.

## Results and discussion

**Fig. 2:**
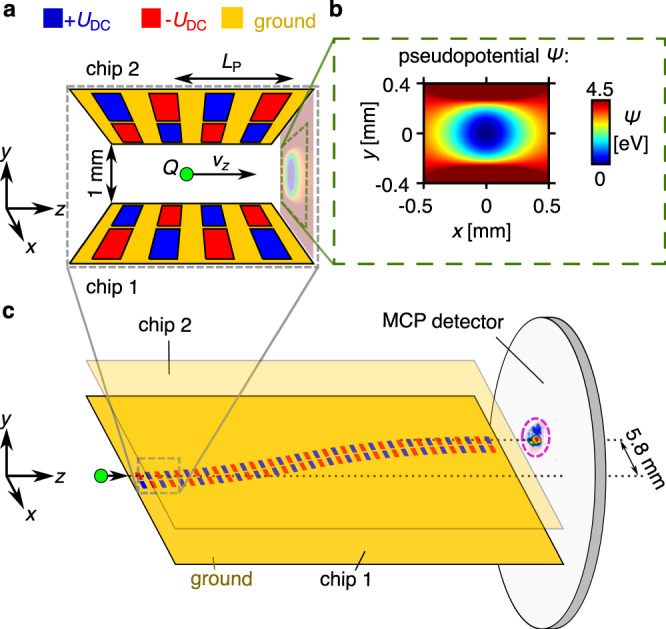
Auto-ponderomotive S-curved guide. **a** A beam of charged particles with charge *Q* and velocity *v*_*z*_ is injected into a guiding structure consisting of two planar chips facing each other with a separation of 1 mm. The chips hold electrodes to which electrostatic potentials +*U*_DC_ (blue) and −*U*_DC_ (red) are applied. Their polarity varies periodically along the structure with the period length *L*_P_ leading to the creation of the guiding pseudopotential for propagating electrons. **b** Simulation of the ponderomotive potential in a cut-plane transverse to the beam for electrons with *U*_DC_ = 100 V and *U*_A_ = −1 kV. The small ellipticity is due to the broken circular symmetry of the planar chips. **c** The electrodes on the chips define an S-curve that guides the particles so that they are laterally displaced. The particles are detected by a microchannel plate (MCP) detector 1 cm behind the structure. For illustration, only the bottom chip 1 is shown in full detail. Chip 2 has the mirrored electrode layout but with inverted polarity as shown in **a**. The detector signal of guided particles is highlighted by a dashed purple circle. A picture of the front and back of the upper chip is displayed in the Supplementary Fig. 2.

Figure 2 shows the design of a guiding structure and the simulation of the auto-ponderomotive potential *Ψ*. Eighty-four electrodes are printed on each of the two chips and define an S-curve with a radius of curvature of *R*_K_ = 0.535 m, such that the output of the guide is laterally displaced by 5.8 mm with respect to its input. Static voltages +*U*_DC_ (blue) and −*U*_DC_ (red) are applied on the electrodes forming a system of quadrupole lenses with spatially periodic polarity (period length *L*_P_ = 5.6 mm). This guide represents the electrostatic equivalent to the curved version of a conventional linear Paul trap with just an alternating potential applied (*α* = 0)^16^. The stability is therefore only determined by the parameter $q = \frac{{\eta L_{\mathrm{P}}^2 U_{{\mathrm{DC}}}}}{{2{\uppi}^2 R^2 |U_{\mathrm{A}}|}}$ (see Supplementary Note 4 and 5). The geometric factor *η* = 0.61 accounts for deviations from the ideal hyperbolic electrode geometry and from the perfectly sinusoidal form of the electric field^23^. *R* = 0.5 mm is the distance from the ponderomotive potential minimum to the chip surface.

**Fig. 3:**
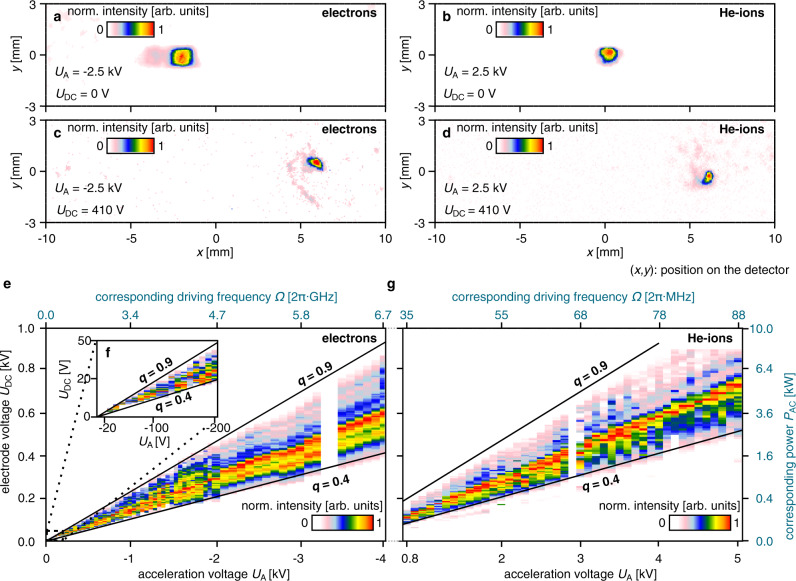
Auto-ponderomotive guiding of various species of charged particles. **a**, **b** Detector images of unguided electron **a** and helium ion **b** beams with electrodes grounded (*U*_DC_ = 0 V). **c**, **d** With charged electrodes (*U*_DC_ = 410 V), both beams are guided and are measured at *x* = 5.9 mm (electrons) and *x* = 6.1 mm (He-ions), almost exactly at the expected position of *x* = 5.8 mm. The barely visible curl structure results from spiraling trajectories of off-centrally injected particles. The detector signal of helium ions is expected to be the vertically mirrored image of the electron signal, due to the opposite sign of their charge, which can just be discerned. **e**, **g** Guiding stability: normalized intensity of guided electrons **e** and helium ions **g** on the MCP detector. For each acceleration voltage *U*_A_, the applied electrode voltage *U*_DC_ was scanned from 0 to 1 kV and the guiding signal of each scan was normalized to its maximum value. For comparison with electrodynamic traps, the corresponding driving frequency and AC power (impedance of 50 Ω) are given on the secondary axes (blue). Black lines corresponding to operation at *q* = 0.4 and *q* = 0.9 are drawn in **e** and **g** as a guide to the eye. Even though their masses differ by more than five orders of magnitude, guiding starts for all particles at *q* ≈ 0.4 and no guiding is observed for *q* values >0.9, perfectly matching our particle tracking results. Because not all kinetic energies were possible to realize due to the source, some regions are left white. **f** Magnified image of a part of **e** showing that electrons are guided for kinetic energies as low as 20 eV, which was the lowest energy we could achieve with our source.

To characterize the guiding stability, we measure the number of guided particles for a range of particle beam energies and electrode voltages. For this, a compact system consisting of a tungsten needle tip, extractor, four deflectors, and two grounded apertures was used as a source of charged particles (see Supplementary Note 1). This enables electron field emission^24^, as well as the generation of an ion beam from background gas^25^ depending on the polarity of the applied acceleration voltage *U*_A_. The charged particle beams are unguided when the electrodes are grounded, as shown in the detector images for electrons in Fig. 3a and for helium ions in Fig. 3b. When voltages are applied to the electrodes, guiding is observed for electrons (Fig. 3c), as well as helium ions (Fig. 3d), evidenced by a shifted detector signal at the expected guide exit position. In contrast to the unguided beams, the position of the guided beams on the detector remains unchanged even when magnets (~1 mT) are brought close to the vacuum chamber.

For each *U*_A_, the applied *U*_DC_ was scanned from 0 to 1 kV. We observe guiding for a large energy range from 20 to 4000 eV for electrons (Fig. 3e, f) and from 800 to 5000 eV for helium ions (Fig. 3g). Even though there are five orders of magnitude difference in the masses, guiding is observed for the same ratios of the applied voltages $U_{{\mathrm{DC}}}/\left| {U_{\mathrm{A}}} \right|$, corresponding to *q* values between 0.4 and 0.9. The lower border in *q* is due to the curvature of the guide. Since the restoring force of this potential depends on *q*, a finite value of *q* ≥ 0.39 is needed to compensate the centrifugal force resulting from the curves (see the Supplementary Note 6), matching perfectly the experimentally observed minimum $q \cong 0.4$. The upper border corresponds similarly well to the maximum *q* value of 0.91 in the first stability region of linear Paul traps^6^. The measurement was repeated for other noble gas ions (neon, argon, krypton, and xenon) yielding similar results (displayed in the Supplementary Fig. 4). For comparison with radio frequency Paul traps, the corresponding driving frequencies and AC powers for an impedance of 50 Ω $\left( {P_{{\mathrm{AC}}} = 0.5 U_{{\mathrm{DC}}}^2/50\;{\mathrm{{\Omega}}}} \right)$ are given on the secondary axes in Fig. 3c, e. Using electrons, the auto-ponderomotive design easily generates an apparent alternating field with driving frequencies in the gigahertz range with tens of kilowatts of AC power, which is virtually impossible to feed or maintain on an electrodynamic chip for thermal load reasons^16,17^. As the stability parameters are independent of the geometrical size, a miniaturized version of the guide will work in the same way and can readily be fabricated on the micrometer scale by lithography. This will lead to higher driving and trap frequencies, for example, using an electron beam with an energy *E* of *E* = 1 keV, a period length *L*_P_ of *L*_P_ = 56 µm and *U*_DC_ = 100 V results in a driving frequency of *Ω* = 2$\pi \sqrt {2E/M_{\mathrm{e}}} /L_{\mathrm{P}}$ = 2π × 0.33 THz and trap frequency of $\omega$ = $q {\it{{\varOmega}}}/\sqrt 8$ = 2π × 46 GHz (with *M*_e_ the electron mass). As the quantum ground state of the transverse guiding potential is described by Heisenberg limited Gaussian profiles in position and momentum space^16^, a Gaussian electron beam in free space perfectly matching the ground state of the guided electrons in its focus will allow for perfect mode-matching (e.g., for electrons with a longitudinal energy of 1 keV, a beam with a width of Δ*x* = $\sqrt {\hbar /2M_{\mathrm{e}}\omega }$ = 14 nm at the focus and an opening angle *α* = $\sqrt {\hbar \omega /E}$ of *α* = 0.4 mrad would be required, which is straightforward to achieve). However, realistic electron beams have an energy width Δ*E* and non-round (e.g., quadrupole) lenses are known to have negative lens aberrations^26^, which is why multipole fields can be used to correct the positive lens aberration of round lenses^22,26^. The energy spread Δ*E* will cause an energy dependent spread of electron trajectories (chromatic dispersion/aberration). As the trap frequency $\omega$ depends on the forward velocity, the profiles of quantum ground state in position and momentum space will vary as well, leading to a lower number of electron in their transverse ground state if the relative energy width Δ*E*/*E* is not sufficiently small. Alternatively, the curve of the guide can be utilized as an energy and mode filter, representing a quantum state-selective element by operating the guide at the minimum *q* value for the slowest electrons, and using the centrifugal force to filter out faster electrons and higher-lying states.

**Fig. 4:**
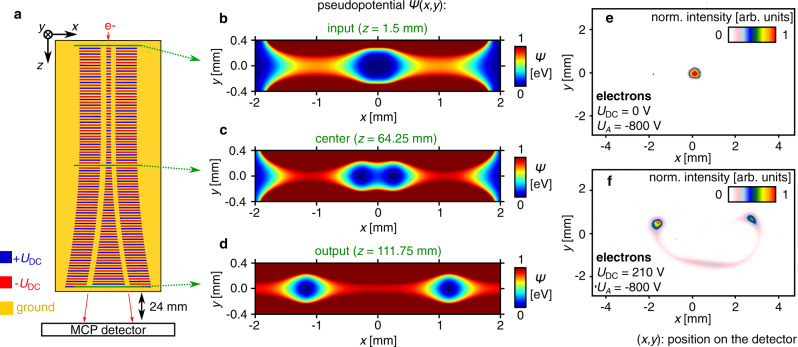
Auto-ponderomotive beam splitting on chip. **a** Top view of the beam splitter chip. The layout consists of three rows of electrodes. The width of the central electrodes widens along the chip from 0.3 to 2.2 mm (not to scale for illustration, see the Supplementary Fig. 3 for a picture of the chip). Like the S-curved guide, the splitter consists of two chips facing each other with a separation of 1 mm. The upper chip 2 has the same electrode layout, but with inverted polarity. The electrode layout is chosen such that the minimum of the ponderomotive potential is continuously split into two minima separated by 2.3 mm at the chip end. To illustrate this splitting, we plot the simulated pseudopotential for an electron beam with *U*_A_ = 800 V and *U*_DC_ = 210 V in the transverse *xy*-plane at *z* = 1.5 mm at the input **b**, at *z* = 64.25 mm **c**, and at *z* = 111.25 mm at the output **d**. Clearly, the initially single central minimum splits continuously into two central minima as the particle propagates down the structure. **e** Detector image of an unguided electron beam (*U*_DC_ = 0 V). **f** Detector image of a split electron beam (*U*_DC_ = 210 V). Two spots are visible with a faint signal of lost electrons in between. The spot distance of 4.2 mm is expected given the opening angle of the split isopotential channels and the detector distance of 2.4 cm.

The next important goal will be to coherently split the ground state of the guided electrons similar to the Y junctions that have been envisioned for ions shuttled in surface-electrode ion traps^11^. Based on the microwave chip-based beam splitter for slow (<200 eV) electrons^27^, we now show the auto-ponderomotive version of an electron beam splitter, which works for electron energies up to 800 eV. The design is shown in Fig. 4a. Here, the electrode layout of each chip consists of 270 electrodes forming three rows. Electrostatic voltages +*U*_DC_ (blue) and −*U*_DC_ (red) are applied, forming a system of multipolar lenses with spatially periodic polarity with a period length *L*_P_ = 2.4 mm. Moving along *z*, the width of the central electrodes widens. This splits the initial central minimum of the ponderomotive potential into two minima, separated by 2.3 mm (see Fig. 4b–d).

A charged particle beam fed into the single central minimum at the structure input splits transversely into two beams following smoothly the auto-ponderomotive potential. This is shown in Fig. 4f for an electron beam (Philips XL30 SEM) with an energy of 800 eV as two distinct beam spots (unguided beam displayed in Fig. 4e). For other electron energies, the voltage *U*_DC_ has to be changed accordingly to maintain the same beam splitting and is only limited by sparking. (A more detailed investigation of the beam splitter will be subject to forthcoming work.) Based on prior quantum mechanical simulations^18^, such a beam splitter miniaturized to the micrometer scale should enable interferometry with guided electrons: by tailoring a smooth transition from a double-well potential to a single minimum back to a double-well potential, and by coupling electrons into the ground state of one arm of the beam splitter, the initial state evolves adiabatically in the ground state of the double-well potential and electrons leave the two output arms of the splitter as two Gaussian output beams separated by only a few µm. As shown recently^28,29^, the vicinity of the metal electrodes should not have any considerable detrimental effects on the coherence of the two output beams. Using the same optical elements like in conventional electron biprism experiments^2^ and a single-atom tip as a fully coherent electron source^30^, both output beams can be shifted longitudinally to restore potentially lost longitudinal coherence^31^, and be recombined, creating interference stripes, which can be made visible on a detector by magnification. Since the relative energy spread of the single-atom sources is very small^32^ at a beam energy *E* of *E* = 1 keV, the spread in the trap frequency is negligible (${\mathrm{{\Delta}}}\omega /\omega$ = ${\mathrm{{\Delta}}}E/2E$) and most electrons can be efficiently injected into their ground state by the direct injection scheme explained above. It should be noted that this beam splitter would act as an amplitude beam splitter for electrons^18^, i.e., the electron-optical analog of a semitransparent mirror for photons, and could enable experiments like interaction-free measurement with electrons^33^. This measurement scheme could be important for electron microscopy, as it is proposed to be used to generate images of beam-sensitive specimens with substantially reduced damage^34^.

Therefore, the here presented devices are not just technically less demanding and more potent alternatives to previously shown planar microwave structures, whose miniaturized version are producible with existing lithographic techniques, they may also herald future experiments entering the frequency range bridging the gap between microwave and optical frequencies, for which ponderomotive forces have been utilized^35–38^, and by that open up an arena for quantum optics experiments and state-selective applications^39^ with free electrons.

## Supplementary information

Supplementary Information

## Data Availability

The raw data, i.e., the recorded detector images, that support the findings of this study are available in Zenodo with the identifier “10.5281/zenodo.4160787”.

## Code availability

The code to reproduce the results is available from the corresponding author upon reasonable request.
