# Supplementary material for: Charged particle guiding and beam splitting with auto-ponderomotive potentials on a chip
Source: Nat Commun. 2021 Jan 15;12:390. doi: 10.1038/s41467-020-20592-4 (PMC7810984; doi:10.1038/s41467-020-20592-4)
Supplement: Supplementary file 1 — Supplementary Information [file 41467_2020_20592_MOESM1_ESM.pdf]

Supplementary Information:

Charged particle guiding and beam splitting with auto-ponderomotive potentials on a chip

Robert Zimmermann[1,2]✉, Michael Seidling[1,2]✉, Peter Hommelhoff[1]✉

[1] Department Physik, Friedrich-Alexander-Universität Erlangen-Nürnberg (FAU), Staudtstraße 1, 91058 Erlangen, Germany

[2] These authors contributed equally.

✉ email: robert.martin.zimmermann@fau.de; michael.seidling@fau.de; peter.hommelhoff@physik.uni-erlangen.de

1. Charged particle gun, electron-optical bench and detector

A compact system consisting of a tungsten needle tip, extractor, four deflectors and two grounded apertures serves as a source of charged particles. The final aperture with a diameter of 400 µm limits the size and the divergence of the beam. The strong fields between the tungsten needle tip and the extractor allow electron field emission[1] as well as ionization of gas molecules[2], depending on the polarity of the applied acceleration voltage. For the ion beams, gas molecules of the desired noble gas species were let into the chamber by opening a leak valve connected to a supply line which was pumped and subsequently flushed by the desired gas. The partial pressure of the gas was in the order of 10^{-6} mbar to achieve a sufficiently intense ion beam at the detector. Tips of varying sharpness and distance to extractor were used to realize particle beams of various energies. For the electron beam splitting experiment, a scanning electron microscope (Philips XL30 SEM), which illuminated a spot (<1 µm) at the input, is used as the electron source. All components are

pre-aligned and fixed rigidly onto a 25 cm long electron-optical bench consisting of two straight ceramic rods. A picture of the setup is displayed in supplementary figure 1. The setup was placed into a vacuum chamber and the charged particles are detected with a microchannel plate (MCP) detector, which is placed 1 cm behind the S-curved guide (2.4 cm after the beam splitter). For the auto-ponderomotive guiding experiment, the measured intensity within a 1 mm wide square at point x = 5.8 mm and $y = 0$ mm on the detector is taken as the signal of the guided charged particles.

2. Layout of the S-curved guide

Pictures of the upper chip's front and back are shown in supplementary figure 2. The S-curved guide consists of two planar chips facing each other with a separation of 1 mm. They are fabricated by a standard printed circuit board process on FR4 substrates with electrodes made from gold-plated copper. The chips have a total length of 11.3 cm and each chip consists of 84 electrodes. The electrodes define an S-curve with a radius of curvature of R_{K} = 0.535 m, such that the output of the guide is laterally displaced by 5.8 mm with respect to its input. The electrodes have a length of 1.3 mm and are 1.4 mm wide. The gap between the electrodes is 100 µm wide. The electrode layout of one of the two chips is displayed in Fig. 2 in the main text. The other chip has the mirrored electrode layout but with opposite polarity. Both chips have countersinks for ruby balls which serve to align the chips. The depth of the countersinks and the diameter of the ruby balls are chosen such that the chips are separated by 1 mm. The S-curved guide can be fixed and aligned to the electron-optical bench with a holder.

3. Layout of the beam splitter

A picture of the electrode layout of one of the two chips is displayed in supplementary figure 3. The other chip has the mirrored electrode layout but with opposite polarity. The beam splitter consists of two planar chips facing each other with a separation of 1 mm. They are fabricated by a standard printed circuit board process on FR4 substrates with electrodes made from gold-plated copper. The chips have a total length of 11.3 cm and each chip consists of 270 electrodes arranged in three rows. The electrodes have a length of 0.55 mm and the gap between the electrodes is 50 µm wide. The outer electrodes have a width of 1.4 mm, while the width of the inner electrodes changes along the splitter from 0.3 mm to 2.2 mm. Alignment and mounting of the chips are done as for the S-curved guides described above.

4. Derivation of the non-relativistic and auto-ponderomotive formula of the parameter *q*

The stability parameter q for a linear Paul trap (with $\Phi_1 = U_{\mathrm{AC}} \cos \Omega t$ and $\Phi_2 = -\Phi_1$ applied to adjacent rod electrodes) is given by $q = \frac{2Q \cdot 2 \cdot U_{\mathrm{AC}}}{M \cdot R^2 \Omega^2}$ with $\frac{Q}{M}$ the charge-to-mass ratio of the charged particles, R the minimal electrodes' distance to the guide center, U_{AC} the amplitude of the alternating potential and Ω the driving angular frequency[3]. To derive the expression of q for auto-ponderomotive guides, one replaces the driving frequency Ω with $2\pi \cdot \frac{v_z}{L_{\mathrm{p}}}$. Here, v_z is the velocity of the charged particles in the beam and L_P the period length of the auto-ponderomotive structure. The velocity depends on the acceleration voltage U_{A} as $v_z = \sqrt{-2 \cdot QU_{\mathrm{A}}/M}$. In our case, U_{AC} must be replaced by U_{DC}. Hence, $q = -\frac{L_{\mathrm{P}}^2 \cdot U_{\mathrm{DC}}}{2\pi^2 \cdot R^2 \cdot U_{\mathrm{A}}}$. Because we use planar electrodes, a geometric factor η needs to be included to describe the effective quadrupole strength of the used geometry[4]. Thus,

the stability parameter is corrected to $q = -\frac{\eta \cdot L_{\mathrm{P}}^2 \cdot U_{\mathrm{DC}}}{2\pi^2 \cdot R^2 \cdot U_{\mathrm{A}}}$ and is valid for non-relativistic particles ($v_z \ll$ speed of light c). The sign of q has no effect on the stability, therefore we only calculate the absolute value. An extension for relativistic velocities can be derived by including length contraction of L_{P} and the Lorentz transformation of the electric field, but the resulting expression is only independent of the particle's charge Q and rest mass M_0 in the limiting cases of relativistically slow and fast particles.

5. Calculating η and the harmonic region of the auto-ponderomotive potential

The ponderomotive potential (also called the pseudopotential in the manuscript) is calculated as $\Psi = \frac{Q^2 \langle E_{\mathrm{F}}^2 \rangle}{4\, M\, \Omega^2}$ with $\langle E_{\mathrm{F}}^2 \rangle$ the time-averaged squared electric field. For auto-ponderomotive structures, $\langle E_{\mathrm{F}}^2 \rangle$ is calculated by the average of the electric field squared along the guide over the period length L_{P}. Compared to the ideal case of hyperbolic electrodes, the field strength of the quadrupole component is reduced by a geometric factor η and is attained by a best fit from simulation. We obtain $\eta \approx 0.61$ for the guiding structure presented in Fig. 2 and Fig. 3 in the main text. Like in any harmonic approximation, the best fit is only valid close to the center. The discrepancy is less than 5% for displacements $\delta x \leq 80$ µm from the guiding center and increases strongly for larger δx.

6. Derivation of the minimum value of q for the S-curved guiding structure

The harmonic force of the ponderomotive potential F_{H} must compensate the centrifugal force F_{Z} to guide the particles in a curve with curvature R_{K}. Stable trajectories are limited to the harmonic region of the guide, where the restoring force $F_{\mathrm{H}} = -\nabla\Psi = -\nabla(\frac{1}{2}\omega^2 M \delta x^2)$ reads $F_{\mathrm{H}} =$

$-\omega^2 M\, \delta x$ with the trapping frequency $\omega = \frac{q}{\sqrt{8}}\Omega$. The centrifugal force is given by the curvature R_K and the particle's velocity v_z. Demanding that $F_\mathrm{H} \geq F_\mathrm{Z}$ leads to the guiding condition $q \geq \frac{L_\mathrm{P}\cdot\sqrt{2}}{\pi\cdot\sqrt{R_\mathrm{K}\cdot\delta x}}$ resulting in a minimum value of q = 0.39 for a guiding structure with the geometry presented in this work, in excellent agreement with the experimentally observed q_min for all guided species (see Fig. 3 in the main text and the supplementary figure 4).

7. Miniaturization leads to higher trapping frequency $\boldsymbol{\omega}$

If the geometry of an auto-ponderomotive structure is scaled down by a factor of c_g, the period length L_P and electrode's distance to the centerline R are reduced to $L'_\mathrm{P} = \frac{L_\mathrm{P}}{c_\mathrm{g}}$ and $R' = \frac{R}{c_\mathrm{g}}$. The driving frequency $2\pi \cdot \frac{v_z}{L_\mathrm{p}}$ increases accordingly to $\Omega' = \Omega \cdot c_\mathrm{g}$. Since the stability parameter $q \propto L_\mathrm{P}^2/R^2$ is independent of c_g, guiding is attained for the same applied voltage ratios for all scaling factors c_g and the trapping frequency $\omega = \frac{q}{\sqrt{8}}\Omega$ increases to $\omega' = \omega \cdot c_\mathrm{g}$. Thus, an electrode layout on the micrometer scale leads to much higher trapping frequencies if operated at the same stability parameter q. For example, using a guide with a period length L_P of 56 µm ($c_\mathrm{g}{\sim}100$) (which is straightforward to manufacture) and an electron beam with a kinetic energy of 1 kV results in a driving frequency of Ω = 2π ·0.33 THz. Operating the guide at q = 0.3 (U_DC = 77.4 V, well below the breakdown voltage of high vacuum) leads to a trapping frequency of ω = 2π · 36 GHz.

8. Extended Data: Auto-ponderomotive guiding for electrons and noble gas ions

The supplementary figure 4 displays the result for all used noble gas ions as discussed around Fig. 3 in the main text. The supplementary figures 5 and 6 show the detector images of guided beams

of various species of charged particles with an acceleration voltage of $|U_\mathrm{A}|$ = 4000 V and the S-guide operated at an q-value operated at q = 0.39 and q = 0.77. Similar to the detector image of the split electron beam in Fig. 4 in the main text, there is, besides the spot of guided particles at the expected spot of x = 5.8 mm and y = 0 mm, a tail of lost particles visible in the supplementary figure 5 for all used charge particle species, which can be attributed to the spiral trajectories of the off-centrally injected particles. Compared to the other charged particles, electrons had the lowest background, as they could be operated at a higher detector gain due to lower gas pressure. The ion beam with the lowest background was realized with helium ions and highest with xenon ions. Since the trajectories of charged particles are vertically mirrored if either the sign of the charge or the polarity of all applied electrode voltages are inverted, the detector image of helium ions in Fig. 5 is the vertically mirrored of the detector image of the electrons and the other noble gas ions which were operated with the polarity of the electrode voltage inverted. Small deviations in the detector signal can be observed as there are small imperfections in the experiment: (1) the quality of the alignment by the electron-optical bench is limited by the fabrication tolerances of the workshop ($\leq$ 30 µm), (2) there is no magnetic shielding, thus especially electrons can be affected by background magnetic field due to their low mass and (3) the precision of the voltage supply is limited by $\sim$1 V.

References and Notes:

1 Crewe, A., Eggenberger, D., Wall, J. & Welter, L. Electron gun using a field emission source. *Review of Scientific Instruments* **39**, 576-583 (1968).

2 Müller, E. W. Field ionization and field ion microscopy. *Advances in electronics and electron physics* **13**, 83-179 (1960).

3 Paul, W. Electromagnetic traps for charged and neutral particles. *Reviews of modern physics* **62**, 531, doi:10.1103/RevModPhys.62.531 (1990).

4 Wesenberg, J. H. Electrostatics of surface-electrode ion traps. *Physical Review A* **78**, 063410 (2008).

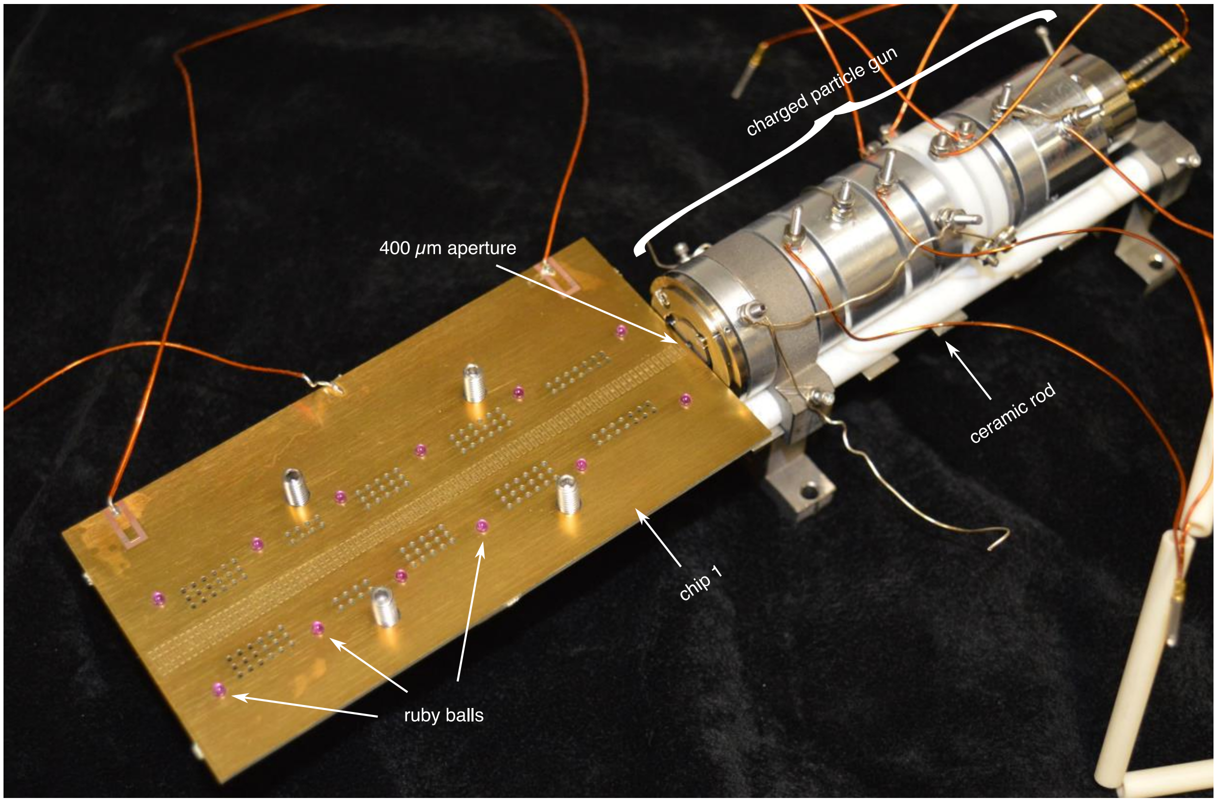

Supplementary figure 1: Picture of the electron-optical bench and charged particle gun. The top chip has been removed for illustration purposes. All components are attached to the electron-optical bench with titanium clamps. The last aperture before the chip with a diameter of 400 μm limits the size and the divergence of the beam. The ruby balls on the chip ensure proper alignment of the two chips.

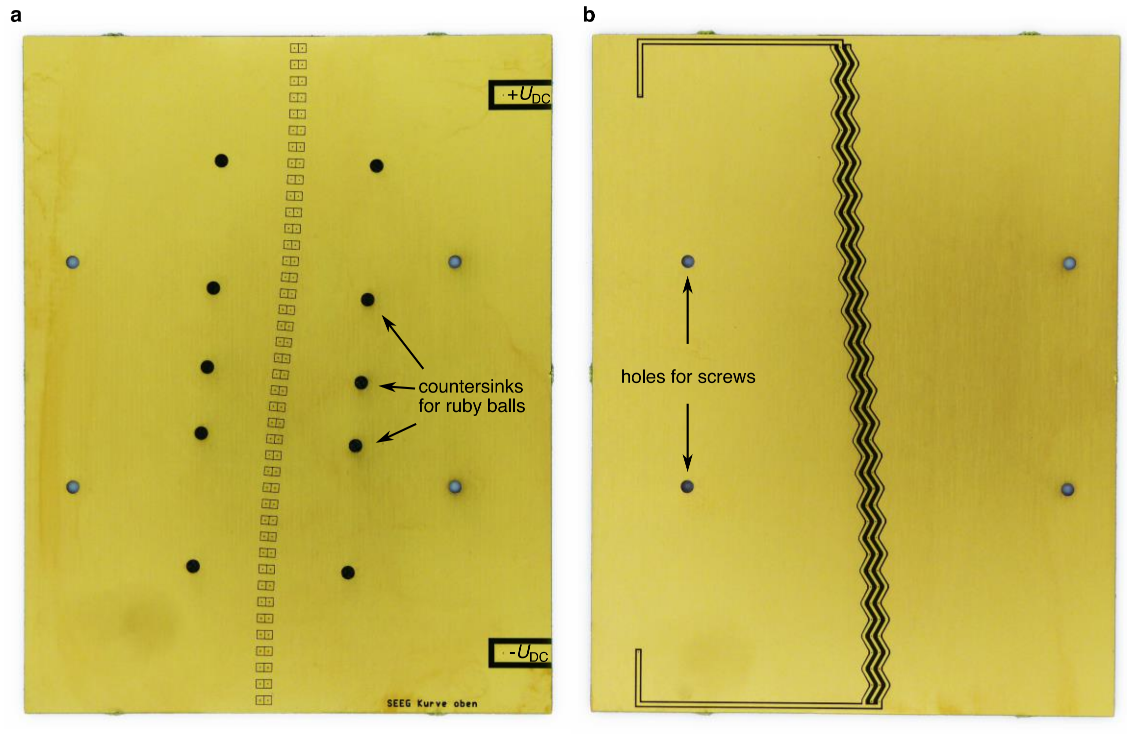

Supplementary figure 2: Front (a) and back (b) of the S-curved guide's upper chip. Meandering electrodes on the back contact the electrodes on the front with plated through-holes (vias) resulting in spatially periodic voltages. The upper chip is placed above the lower chip such that their front sides are facing each other. Countersinks for ruby balls and holes for screws are drilled for alignment and fixation.

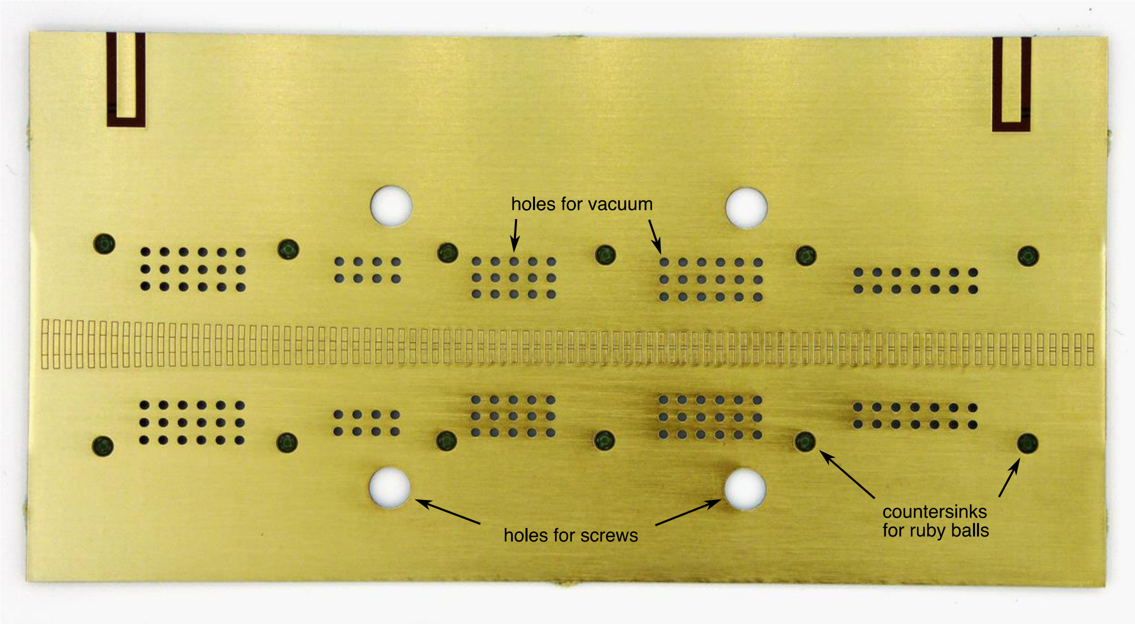

Supplementary figure 3: Front of the beam splitter's chip. 270 electrodes arranged in three rows. The width of the inner electrodes changes along the splitter from 0.3 mm to 2.2 mm, while the width of the outer electrodes is 1.4 mm. Countersinks for ruby balls and holes for screws are drilled for alignment and fixation. Additional smaller holes are used to ensure good vacuum.

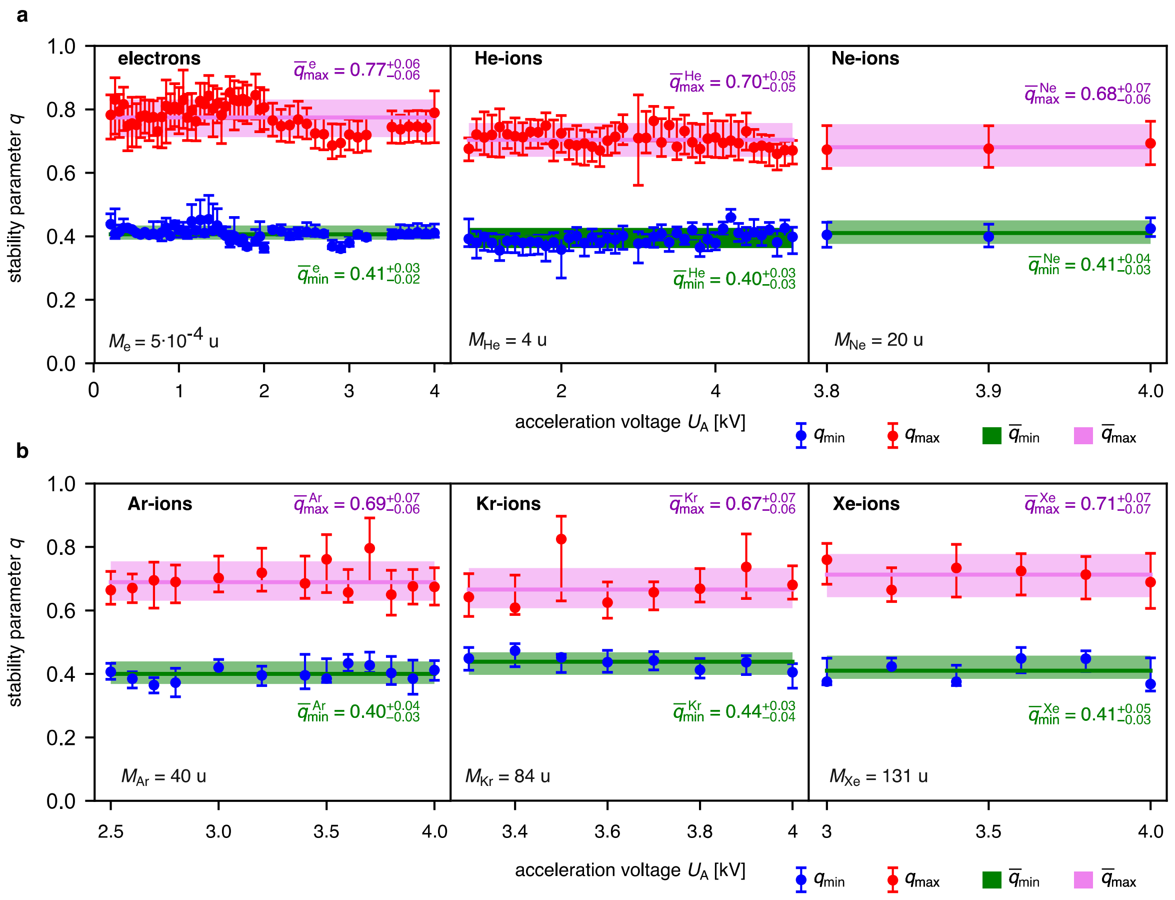

Supplementary figure 4: Auto-ponderomotive guiding of various species of charged particles. **a, b** The maximum and minimum q values (defined as q at 1/e the maximum intensity) for electrons and all used noble gas ions calculated from the detector intensity of the guided charged particles obtained by voltage scans (as discussed in the main text around Figure 3). As the guiding signal, the intensity within a 1 mm wide square at point $x = 5.8$ mm and $y = 0$ mm on the detector is taken. The dependence of the guiding signal on the appied electrode voltages was fitted by a positively skewed gaussian distribution and the error bars indicate the uncertainty of the 1/e position. Note that the mass and charge-to-mass ratio varies over more than a factor of 200 000, clearly demonstrating that auto-ponderomotive potential engineering works independently of them. The

measured maximum values of q vary stronger than the minimum values because guiding becomes more sensitive to imperfect injection for high q as the acceptance phase-space for coupling the beam into the guide decreases.

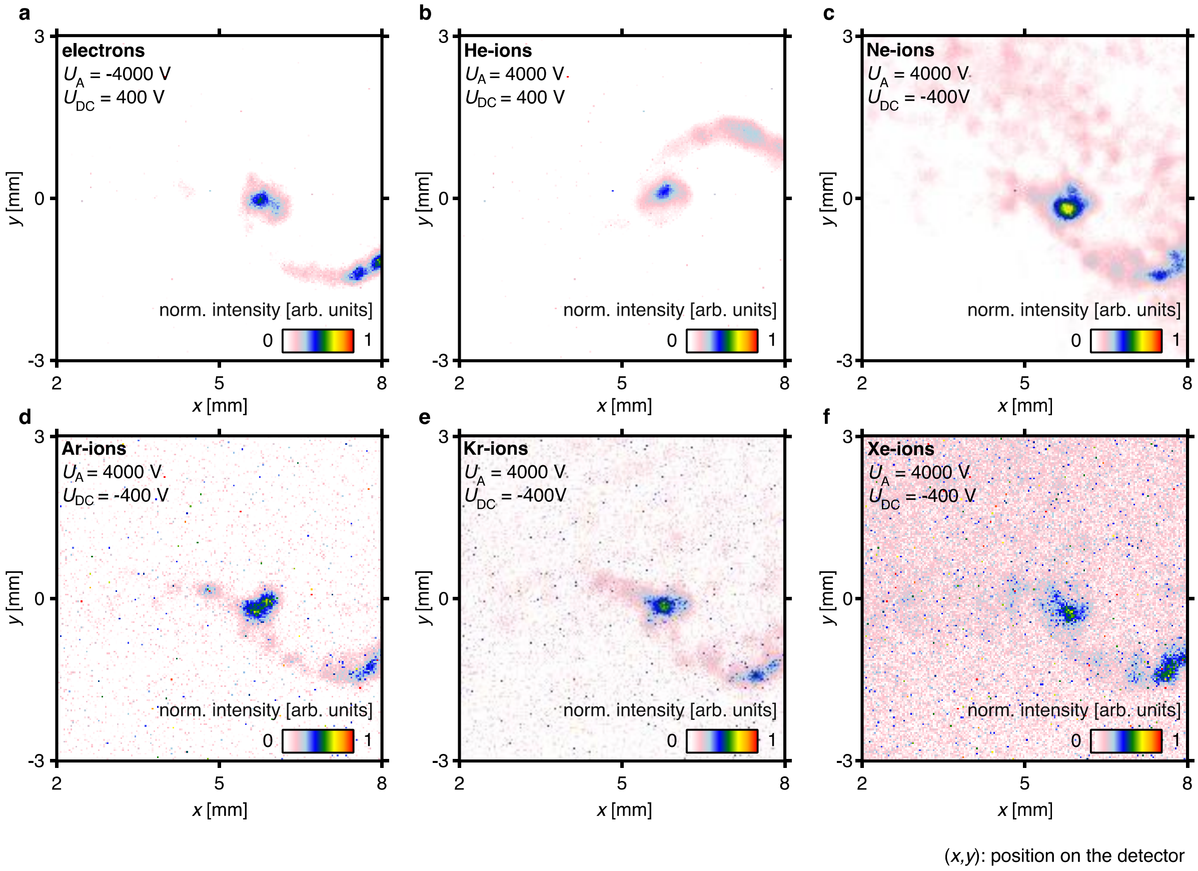

Supplementary figure 5: Detector images at $q = 0.39$ for various species of charged particles. The spot at around $x = 5.8$ mm and $y = 0$ mm can be attributed to guided charged particles, while a tail of lost particles is visible. The curly structure of the tail can be explained by the spiral trajectories of the off-centrally injected particles. Comparing the detector images of the electrons (**a**) and helium ions (**b**) with each other, the detector signal of helium ions is in good approximation the vertically mirrored image of the electron signal. This is (as explained around Fig. 3 in the main text) due to the opposite sign of their charges. The detector image is also expected to be mirrored vertically if the polarity of the applied electrode voltages is inverted. Thus, the detector images for the neon (**c**), argon (**d**), krypton (**e**) and xenon ions (**f**) are qualitatively the same as the detector

image of the electrons despite the opposite sign of their charges, however, the background is much higher than compared to the electron or helium ion beams.

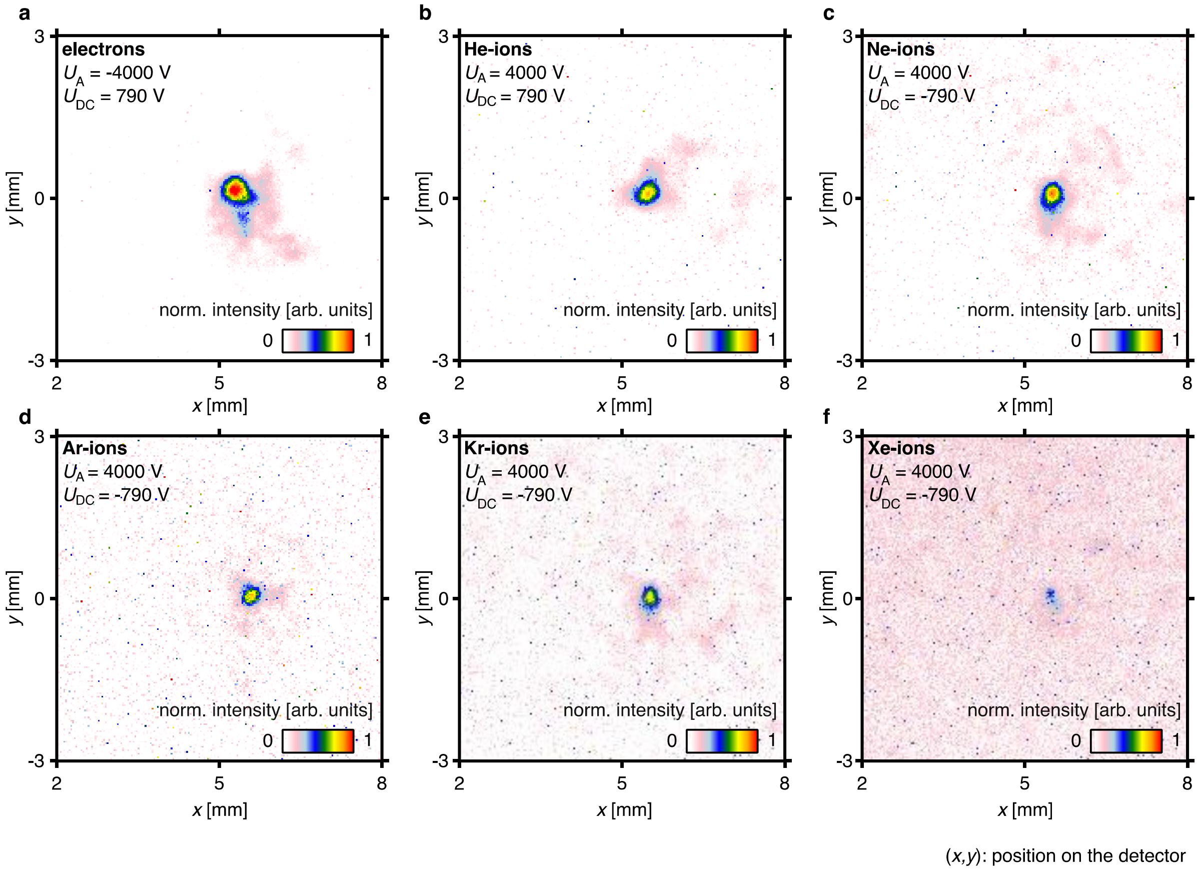

Supplementary figure 6: Detector images at q = 0.77 for various species of charged particles. A signal of guided particles is visible at around x = 5.3 mm and y = -0.1 mm for electrons (**a**) and at around x = 5.5 mm and y = -0.1 mm for the other charged particle species. This small discrepancy in position is due to small imperfections in the experiment: (1) alignment is not ideal due to fabrication tolerances, (2) background magnetic field and (3) limited precision ~1 V of the voltage supplies. Like in Fig. S5, the background of the neon (**c**), argon (**d**), krypton (**e**) and xenon (**f**) ion beams is higher than compared to the electron or helium (**b**) ion beams.
